# Recruitment issues in a multicenter randomized controlled trial about the effect of the Cultural Formulation Interview on therapeutic working alliance

**DOI:** 10.1016/j.conctc.2024.101373

**Published:** 2024-09-27

**Authors:** Alma M. Brand, Simon P.N. Groen, Samrad Ghane, Nathalie Destoop, Hannah E. Jongsma, Bernard G.C. Sabbe, Özlem Becan, Dhiya Alyan, Mario H. Braakman

**Affiliations:** a Tilburg Universiteit, Tilburg, Netherlands; b De Evenaar, Centrum voor Transculturele Psychiatrie, GGZ Drenthe, Beilen, Netherlands; c Parnassia Groep, Utrecht, Netherlands; d Psychiatrisch Ziekenhuis Alexius van Grimbergen, Brussel, Belgium; e Centrum voor Transculturele Psychiatrie Veldzicht, University Center of Psychiatry, University Medical Center Groningen (UMCG), Balkbrug, Netherlands; f Universiteit van Antwerpen, Antwerpen, Belgium

## Abstract

This short communication concerns recruitment issues in a multicenter randomized controlled trial. An overview of anticipated and unexpected recruitment issues at various organizational levels is discussed as encountered in this trial. These experiences are shared to assist researchers in avoiding similar experiences, prevent wasting valuable research resources, and justify the time and energy committed by enrolled participants.

## 1 Introduction

A multicenter randomized controlled trial (RCT) was initiated to examine the effect of the Cultural Formulation Interview (CFI) on therapeutic working alliances mediated through perceived cultural empathy among first or second-generation migrant patients [1]. A two-year ZonMw grant was received to support this research, which required a sample size of 164 participants for statistical power. Recruitment issues have been identified as significant threats to the successful conduct and reliability of RCT results. Various factors may threaten the recruitment process, resulting in delays or early termination, which may introduce bias, limit generalizability, and compromise the representativeness of the target population [[2], [3], [4]]. Underestimation and unawareness of recruitment issues are common when designing an RCT protocol, particularly when the context, population, and topic are new [2,4]. Researchers frequently underestimate the feasibility of recruiting the requisite sample size within the allotted time, which can result in a lack of statistical power [2]. This brief communication reflects on the recruitment issues encountered in this multicenter RCT to assist other researchers in avoiding similar issues and preventing the waste of valuable research resources. The ethical concerns regarding the time and energy committed by enrolled participants further justify the need to disseminate information regarding the recruitment issues encountered.

## 2 Principal investigator

Recruitment of a principal investigator was problematic. Months of advertising yielded one applicant, reflecting low numbers of available postdoc researchers and research opportunities in Dutch mental healthcare. This delay delayed organizing approvals and reduced time for patient recruitment. Using the University and other academic recruitment services, LinkedIn, and researchers’ professional networks might help recruit appropriate candidates sooner.

## 3 Anticipation

Timely informing eligible Board and management teams about the intended trial and discussing approval requests is important because initiated participation in other research projects may compromise data collection resources and approvals [3]. The unexpected retraction of the Board approval at one location and the reluctance of team managers concerning the anticipated clinician-related unaccounted time and finances per patient treatment trajectory resulted in delays of many months. A budget to cover or share the costs of unaccounted time with the participating center was available but this may not have been communicated enough. Prospective research examining the long-term clinical and financial benefits of implementing clinical interventions may facilitate this approval process. Additionally, the trial design could consider the integration of interventions into patient-accounted time and standard care procedures to facilitate their implementation. To be able to invite additional mental health centers for participation, ethical permission was requested and granted, under the condition that identical procedures were followed at all times.

## 4 Predictability

The low approval and recruitment result might have been better anticipated if Patient and Public Involvement and Engagement (PPIE) were explored to better estimate the to-be-expected involvement, engagement, and participation of boards, managers, clinicians, and migrant outpatients, time and financial investments in this trial [4,5]. In PPIE, the SEAR (screening, eligibility, approach, and randomization) framework could support the assessment of recruitment issues tailored to the characteristics of intended trials [6]. The optimism of recruiting sufficient migrant outpatients by affiliations unjustifiably disqualified thorough PPIE exploration. PPIE could have also helped to better determine the target population and might have provided a more robust basis for excluding inpatients and outpatients already receiving treatment. Inpatients were excluded, given the higher complexity of their psychiatric problems, and concerns about their ability to adequately communicate their visions and treatment needs. Their inclusion could have provided additional insight into the influence of CFI use in the longer-established therapeutic alliances between patients and clinicians. Furthermore, an up-to-date overview of available SEAR-logged trial experiences might have provided valuable information for the planning and conduct of this trial.

## 5 Affiliation

The principal investigator's lack of affiliation with and distance from the participating locations limited the monitoring and support of recruitment. Furthermore, the in-person data collection among migrant outpatients was anticipated to be time-consuming, necessitating extensive travel. Consequently, the intention was to appoint research assistants and experts by experience at each participating mental health center to assist with recruitment and data collection. Unfortunately, only one research assistant was recruited due to others preferring to participate in other research projects, one researcher left for another position resulting in collaboration problems and delayed approvals. Due to the delays and limited inclusions two of the four involved experts by experience dropped out. The lack of available assistants and the temporary withdrawal of researchers for personal reasons resulted in further delays and highlighted the necessity of a contingency plan. A solution could involve collaboration with available on-site experts by experience.

## 6 Recruitment

Clinicians were asked to recruit newly registered migrant outpatients during intake sessions, provide information about participation, and inform the principal investigator about eligible participants. However, due to staff shortages, long waiting lists, a slow inflow of new patients, high caseloads, trained clinicians dropping out for new positions, and part-time employed clinicians, the commencement of new treatment trajectories and the recruitment of migrant outpatients were delayed and constrained. Despite clinicians’ best efforts, not all inclusions resulted in the collection of complete data sets. Due to the prioritization of urgent care over participation, some undesirable longer-established therapeutic relationships resulted in the exclusion of some migrant outpatients [1]. Furthermore, included migrant outpatients discontinued their participation due to changes in their migration status, which resulted in the termination of their care trajectories. Early termination of treatment trajectories is common among migrant patients and was anticipated [7,8].

## 7 Dedication

Anticipated or perceived participation burdens, lack of interest or need for help, previous negative experiences with participation in scientific research, and lack of confidence in interventions are known recruitment issues among potential research participants [4]. In this trial, clinicians were required to familiarize themselves with the CFI as an intervention. To enhance confidence in the use of the CFI, clinicians had to follow a 2-h training session about the administration of the CFI, similar to the field trial CFI training before being permitted to recruit migrant outpatients [9]. However, enrollment and scheduling of training sessions for the clinician teams proved a time-consuming challenge. To train as many clinicians as possible, motivated clinicians were offered the opportunity to participate in available training sessions. The reliance on a single researcher for the provision of the training converted into an unexpected recruitment issue when scheduled training sessions had to be canceled and postponed due to personal circumstances. This subsequently resulted in enrollment delays for migrant outpatients. In future studies, a backup trainer needs to be appointed to avoid delays for this reason. Despite the training, inexperience and the limited time to familiarize themselves with the content of the CFI might have influenced clinicians' motivation to enroll migrant patients. A pilot study that includes a fidelity and risk assessment on reasons for abstinence despite motivation, addressing clinicians’ feelings of competence, available time and energy, worry about work schedule disruption, and willingness to adhere to the study protocol, including qualitative evaluations might have been informative.

## 8 Comprehension and confidence

Suspicion toward participation in research and language barriers for which interpreters were used might have influenced migrant patients' motivation to participate [10,11]. To address some language-related issues, a webpage was developed and published to provide participants with download links to written, auditory, and visual information in different languages. However, the comprehension of the abstract concepts of empathy and working alliance remained problematic for some migrant outpatients and some were suspicious about the exchange of collected information by researchers and clinicians. Funding to translate information letters comprehensively and questionnaires validly and reliably into more than seven languages may have enhanced migrant outpatients’ comprehension and motivation for participation and reduced the need for interpreters. PPIE and qualitative evaluations are known to provide valuable insights into motivations and experiences associated with potential barriers to participation in large trials. This trial may not have been estimated as large enough for the funder to request PPIE, and the need for PPIE and qualitative evaluations was underestimated by the researchers. Table 1 shows the recruitment and approval overview and by which parties these were arranged in this trial.

**Table 1:**
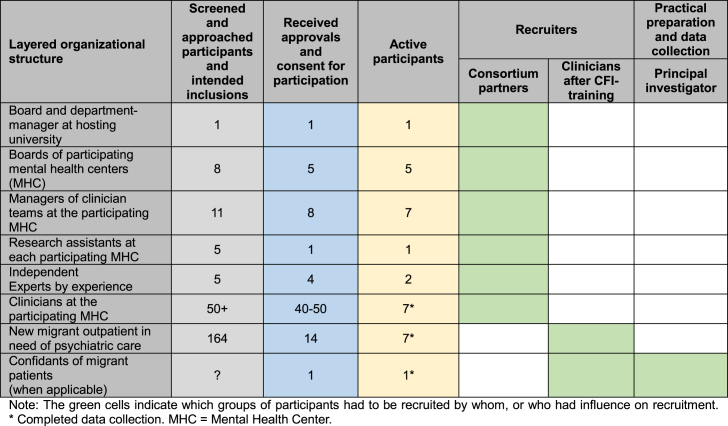
Recruitment, approval, and inclusion overview in the multicenter randomized controlled trial on the influence of the Cultural Formulation Interview on therapeutic working alliance.

## 9 Conclusion

It is prudent to anticipate and arrange all necessary financial and practical approvals before the start of an RCT to prevent delays and the waste of valuable time and money. A pilot study, including a fidelity and risk assessment, PPIE using a SEAR log, and qualitative evaluations are strongly recommended in complex multicenter RCTs. It is crucial to pre-schedule dates for the training of participants who need to assist in the recruitment process and conduct the intervention. It is recommended to allow clinicians time to familiarize themselves with the intervention and to arrange on-site recruitment support to optimize the chances of conducting an RCT successfully.

## CRediT authorship contribution statement

**Alma M. Brand:** Writing – original draft, Conceptualization. **Simon P.N. Groen:** Writing – review & editing. **Samrad Ghane:** Writing – review & editing. **Nathalie Destoop:** Writing – review & editing. **Hannah E. Jongsma:** Writing – review & editing. **Bernard G.C. Sabbe:** Writing – review & editing. **Özlem Becan:** Writing – review & editing. **Dhiya Alyan:** Writing – review & editing. **Mario H. Braakman:** Writing – review & editing, Supervision.

## Declaration of competing interest

The authors declare that they have no known competing financial interests or personal relationships that could have appeared to influence the work reported in this paper.

## Data Availability

No data was used for the research described in the article.
